# Gender Inequality and the Sexual and Reproductive Health Status of Young and Older Women in the Afar Region of Ethiopia

**DOI:** 10.3390/ijerph17124592

**Published:** 2020-06-26

**Authors:** Muluken Dessalegn, Mhiret Ayele, Yeshitila Hailu, Genetu Addisu, Sintayehu Abebe, Haset Solomon, Geteneh Mogess, Virginia Stulz

**Affiliations:** 1 Amref Health Africa in Ethiopia, Monitoring, Evaluation and Research Department, Addis Ababa P O Box 20855 Code 1000, Ethiopia; Mihret.Ayele@Amref.org (M.A.); Geteneh.Moges@Amref.org (G.M.); 2 School of Nursing and Midwifery, Western Sydney University, Sydney, NSW 2751, Australia; v.stulz@westernsydney.edu.au; 3 Amref Health Africa in Ethiopia, Head of Programs, Addis Ababa P O Box 20855 Code 1000, Ethiopia; YESHITILA.HAILU@Amref.org; 4 Amref Health Africa in Ethiopia, Reproductive Maternal and Child Health Department, Addis Ababa P O Box 20855 Code 1000, Ethiopia; Genetu.Addisu@Amref.org (G.A.); Sintayehu.Abebe@Amref.org (S.A.); haset.solomon@gmail.com (H.S.)

**Keywords:** gender, gender analysis, gender equity, value, absuma, empowerment

## Abstract

The main purpose of this research was to analyze gender context in the Afar region of Ethiopia and propose a set of strategies or actions to improve adolescent and youth health. Using a pre-established gender analysis framework, an explorative qualitative study was conducted in five districts. Sixteen key informants and eight focus group discussions were conducted among adult women and men of young adolescents and youth. The study revealed that younger and older women are the most disadvantaged groups of the society. This is due to the high workload on women and girls (housekeeping, building a house and taking care of cattle and children), they also are less valued, have no control over resources and have no part in decision making, including their personal life choices. As a result, they rarely access school and health facilities. They are forced get married according to arranged marriage called “absuma.” As such, they suffer from multiple reproductive health problems. Women have poor decision-making autonomy, lack control over resources, have limited participation in socio-economic practices, and experience child and early forced marriage, and this poor service utilization has exposed them to the worst sexual and reproductive health outcomes.

## 1. Introduction

Achieving the third and fifth United Nations (UN) Sustainable Development Goals (SDGs) to reduce maternal morbidity, mortality, and gender inequality by the year 2030 are amongst the most pertinent targets for women’s health [1]. Achieving universal health coverage, including access to essential health care services by 2030, has been a top priority for developing countries. Moreover, the 2030 Agenda promises to put an end to barriers that prevent younger and older women from realizing their full potential [1,2]. Despite the numerous development programs that promote women worldwide, disparity in health and many other gender-related indicators remain at stake and significant challenges lie ahead. High morbidities and mortalities remain the challenges of health care programs and policies/strategies, particularly in low-income countries [1,3]. 

Ethiopia has demonstrated significant progress in sexual and reproductive health (SRH) outcomes over the last two decades. Notable achievements have been made in a wide range of health indicators including increased rate of contraceptive prevalence (CPR) from 3% to 36% and reduction in the total fertility rate from 7.7 to 4.6 children between the 1990s and 2016 [4]. However, the progress is not uniform across regions and population groups. While the national CPR is a little over 50% amongst women in urban areas, a vast number of women in the lowest wealth quintile and adolescents and women from majority pastoralist regions (including Afar) still do not receive any form of family planning [4]. 

The points of transition over life stages are always changing and evolving. In Ethiopia, for example, over the course of life, several women and their babies die in childbirth, a young person would often be working before 16 years of age, and adults are considered elderly in their late 40s. Legislative changes should focus on the importance of school education and better preparation for retirement. Understanding child development is crucial and the importance of the very first years of life for normal neurological development. The importance of the wider determinants of health, such as employment and housing, have provided a much greater level of granularity across the life-course [4].

The Afar region remains one of the regions in Ethiopia with poor sexual reproductive health (SRH) indicators. Human immunodeficiency virus (HIV) and sexually transmitted infection’s (STI’s) prevalence among the reproductive age group is high (2% and 0.5%, respectively) and only half of the women (50.7%) received antenatal care (ANC) at least once, which is less than the national average [5]. The region has the second lowest skilled delivery assistance rate (16%) in Ethiopia next to the Somali region (18%). This implies that 84% of births occurred at home without close supervision by a skilled provider. The region has the second highest total fertility rate (5.5) next to the Somali region (7.2), the highest rate of teenage childbearing, and the lowest proportion of women who want to limit childbearing at 12% and a contraceptive prevalence rate of 11.6% [5]. Almost all (98%) women aged 15–49 years of age have undergone either one or more forms of female genital mutilation/cutting (FGM/C).

Nearly one in five women in Afar reported being in a polygamous union and 11% of men have two or more wives. Child marriage is a widespread practice in Afar contributing towards the lowest median age of the first marriage being 16.4 years of age, with the lowest median age at the first time of sexual intercourse being 16.2 years of age for women aged 25–49 years in the country. The literature has shown that babies born to mothers under the age of 20 face a higher risk of being stillborn or dying in the first few weeks of life compared to those born to mothers aged 20–29 years of age [6]. Young women are also more likely to have low birth weight babies, which poses risks of long-term developmental effects [7]. In Afar, pregnancy and childbirth complications are the leading cause of death in women aged 15 to 19 years [7]. Early childbearing may also have negative social and economic effects on women, their families and communities. Young women who commence childbearing often do not complete secondary school education, limiting their future employment possibilities and other life choices throughout the course of their lives. 

Despite the scope of the problem in women’s health, there were limited studies that identified the gender context in Afar that might correlate with such critical health problems. Therefore, the aim of this study was to explore the gender dynamics and inequality that contributes to low reproductive health service utilization and high negative sexual and reproductive health (SRH) outcome indicators in Afar, Ethiopia. 

## 2. Materials and Methods 

### 2.1. Study Setting, Design and Population

According to the Central Statistical Agency (CSA), the total population of the Afar regional state is estimated to be about 1.9 million in 2019 with a male to female ratio of 1:21. This reveals a disproportionately higher number of males than females in the region, unlike other regional states in Ethiopia [8]. Approximately 81% of the population are living in rural areas and nearly 90% are pastoralists which makes them mobile, so they are travelling to different places in different seasons and are nomadic in nature. Ninety-one percent of people originated from the Afarri ethnic group and 96% are Muslims by religion [9].

Arable land constitutes only 5.2% of the total land area. Two-thirds of the region (66%) is degraded and rocky which makes the land unsuitable for cultivation and grazing, exacerbating food insecurity and ultimately resulting in a high prevalence of malnutrition and related health problems in the region. Moreover, the Afar region is frequently affected by persistent drought and is classified as one of the drought-affected regions in Ethiopia. Rainfall is erratic and scarce. The region’s altitude ranges from 1600 m above sea level to 116 m below sea level [10]. Temperature varies from 25 ℃ during the wet season to 48 ℃ during the dry season. 

The Afar people practice a traditional Clan administration system. Clan is the lowest structural unit in the traditional administration system on which communal property rights rest, including land-ownership rights and use of natural resources [11]. Each Clan has its own territory. The boundaries are usually marked by landscapes and some naturally occurring water outlets such as rivers and lakes. Often, the boundaries restrict certain Clans, whilst others may cross the boundaries. Clan territories often comprise strategic resources such as grazing areas (as most Afar people are pastoralists), including dry season evacuations and water points [12]. 

This research employed an explorative qualitative study design. The study was conducted amongst five districts (Woredas) in Zone 3 of Afar, namely: Amibara, Argoba, Awash Fentale, Awash town, and Dulecha. The study districts were selected randomly from Zone 3 and the sample size was determined using the point of saturation for qualitative data analysis. The sampled districts were predominantly rural in nature. The majority of focus group discussion study participants lived in rural areas and a few key informants lived in semi-urban areas. 

Key informant interviews and focus group discussions were chosen as methods of exploring the gender context in Afar. Most of the sector offices had an understanding of gender in one or other way. The study population included adolescents and youths aged 10–29 (both in and out of school), adult women and men aged 30–49 years of age and 50–54 years of age, respectively, SRH service providers from selected health facilities, and officials and experts from selected Woreda sector offices including Health, Education, Social Affairs, Youth and Sport, and Women’s Affairs offices. Desk reviews were also conducted to explore the legal frameworks of the country in relation to sexual and reproductive health including gender-based violence and harmful traditional practices such as female genital mutilation (FGM). 

### 2.2. Data Collection Process

Data were collected from December 2018 to January 2019. An interview guide was developed and adopted using the different gender analysis framework dimensions after reviewing the literature to guide the interview for both key informants and focus group discussion study participants. A total of 16 key informant interviews and eight focus group discussions were conducted. Focus group discussions were formed in such a way that homogeneity was maintained. Each focus group discussion session had members ranging from six to 13 individuals. The focus group discussions constituted male and female only: adolescents aged 10–14 years of age, young people aged 15–19 years of age and 20–29 years of age, adults aged 30–49 years of age and 50–54 years of age. Besides maintaining the homogeneity of the focus groups by gender and age, participants were also grouped by their schooling status (i.e., having groups of in- and out-of-school adolescents) and marital status.

Focus group discussions and key informant interview guides or discussion facilitation protocols were developed prior to the fieldwork to avoid information collection bias. Both focus group discussions and key informant interview tools were translated into the Amharic language. Key informant interviews were conducted to complement the focus groups and to further explore the research questions related to gender, sexuality, and reproductive health of adolescents and women. Key informant interviews were also used to inquire about the perspectives on gender issues of the local government officials and healthcare service providers that were not reached through focus group discussions. The digitally recorded interviews were transcribed and translated into English.

### 2.3. Qualitative Data Analysis

The qualitative data analysis involved thematic coding of transcribed and translated in-depth interviews and focus group discussions. A hybrid coding approach, which includes the process of creating pre-set and emergent codes, was used. Ideas, concepts, actions, relationships, and meanings that evolved from the data were different from the pre-set codes used as the emergent codes. Data were then analysed using a thematic approach by conducting an ongoing content analysis. Emerging themes were developed from the expanded interviews and discussions. In general, the qualitative data analysis followed the five interrelated steps: reading, coding, displaying, reducing, and interpreting. ATLAS.ti 8 windows (Ethiopia) was used to analyse all the qualitative content. 

### 2.4. Gender Analysis Framework (GAF)

We used multiple gender analysis frameworks (GAF) to conduct this gender analysis. The existing GAFs include the Harvard Analytical Framework, the Moser Gender Planning Framework, the Gender Analysis Matrix (GAM), and the Women’s Empowerment Framework which varied in terms of depth of factors, timing, set-up, focus (social, political, etc.), and other scopes of analysis. In this gender analysis, considering the scope of analysis, type of information needed, and the social, economic, religious, and political context of the study area, we used a combination of formal frameworks [13,14]. We employed two phases of gender analysis. In the first phase, we analyzed the four most important interconnected domains of change in gender relations and strategies as outlined in almost all of the formal gender analysis frameworks. In the second phase, based on the identified domains of change in the first phase, we analyzed factors on gender differences and identified the most prominent gender considerations and indicators of gender inequity. 

The different dimensions considered during the gender analysis are briefly presented as follows: 

***Dimension 1***: Gendered division of labour and workloads. In this dimension, we examined the different roles and responsibilities for adolescents and youth and how this affects their ability to access sexual and reproductive health information and services. This dimension also examined how political, economic, and social systems structure the gendered division of labour within households and communities.

***Dimension 2***: Access and control over resources, assets and services, and household and community level decision-making. This dimension examined the power of younger and older men and women and the strength of power they control and exercise over resources at household and community levels. This dimension explored interactions between young and older men and women at household and community levels that might affect who has access to benefits and resources. In addition, the benefits of controlling resources and the types of resources that younger and older men and women can access inside and outside of the household were explored. The effect of such access to resources on the sexual and reproductive health of adolescents was also analysed as part of the analysis of gender factors in phase two of this study. 

***Dimension 3***: Younger and older women’s sense of self-efficacy and ability to make life choices. This dimension mainly focused on younger and older women’s decision-making power on issues that affect their lives including awareness and ability to use sexual and reproductive health services. It covered adolescent and youth social, psychological, emotional, and political assets, values, and the strategies they create to satisfy their practical needs and strategic interests in relation to sexual and reproductive health rights in the community.

***Dimension 4***: Laws, Policies, and Institutions. Different legal rights affect the capacity of each gender to access services or resources and make decisions based on existing policies and institutions. In this gender analysis, we reviewed national and local documents with respect to gender. Based on the above dimensions, we analyzed factors affecting gender relations. We further analysed the effects of the gender inequalities and correlations associated with key women’s reproductive health issues.

### 2.5. Ethical Considerations

Ethical approval was obtained from the Research Ethics Review Committee (RERC) of Samara University’s Health Science College (ETCO/Admin/00979/17). Participants were informed about the nature and purpose of the study, the methods, and how the results would be used before their informed consent was obtained. Participants were also informed that withdrawal from the study at any time would not have any consequences. In the case of adolescent participants, consent was sought from their parents, partner or legal representative, although the majority of participants were older than 18 years of age. The confidentiality of the data was guaranteed by preserving the anonymity of the study participants and the data were de-identified. Privacy was ensured by conducting the interview in a communally conducive environment.

## 3. Results

The findings of the study are presented in line with the dimensions used in the gender analysis framework. We have also provided background information about the legal and policy frameworks related to gender. In addition, the first section showed the demographic characteristics. 

### 3.1. Demographic Characteristics of Study Participants

For focus group discussions, adolescent and youth, adult men, and women of childbearing ages and those who had experienced pregnancy and/or childbirth were included in the focus group discussions. Study participants were aged between 10–54 years of age (Table 1). The homogeneity of the focus group participants was maintained by marital status (married vs. never married), age (adolescent and youth, adult), schooling (in-school and out-of-school adolescents and youth), and gender (males vs. females) (Table 1).

### 3.2. The Legal and Policy Frameworks Related to Gender

The 1994 constitution of Ethiopia promotes the equality of men and women in the socio-economical, legal and political system. The constitution grants equal citizenship rights for both men and women; automatically forbids gender-based discrimination; affirms women’s equal rights and decision-making power during marriage and divorce; and more specifically grants affirmative action for women as compensation for historical discrimination. The constitution also addresses the state’s obligation for eliminating traditions or norms that harm women mentally or physically [15].

The 2000 revised family law of Ethiopia placed the legal age of marriage at 18 years or older. The law also grants equal rights for women to select their family residence and family administration including decisions related to family property [16]. The 2004 Penal Code of Ethiopia protects violence against women. The new Code states, “Sexual violence against women and minors, and harmful traditional practices (HTPs) such as female genital mutilation (FGM), and early marriage and abduction [including with intent to marry] practices to which women and girls are especially vulnerable are punishable by law” [17]. 

Assurance of healthcare for all segments of the population is also one of the top priorities of the Ethiopian Health Policy, which states that special attention shall be provided to the health needs of women and children, among others [18,19]. The promotion of women, youth, and other vulnerable segments of the population received significant attention in Ethiopia’s Growth and Transformational Plan (GTP), which is a key step towards achieving the country’s development goals [20].

As stated above, the Ethiopian government does not support gender inequality including harmful traditional practices and gender-based violence. Regional states, including Afar, adopted the national legislative provisions and frameworks. The regional government of Afar has created a structural agreement in the government office to lead gender-related issues against women and children [5,19]. However, the findings of this study showed that government offices have limitations in terms of rolling out the desired plan to respond to the needs of women in the region. This has resulted in poor resources and reduced capacity to protect the rights of the Afar people. In addition, this study found that government offices do not demonstrate a unified approach and there is a need for an urgent, integrated response to produce resources for the Afar region, otherwise women will continue to experience challenges in the Afar region [4,5]. 

### 3.3. Gendered Division of Labour and Workload

The study results show that younger and older women from Afar take on significantly more unpaid in-house and external duties and responsibilities as compared to younger and older men. Most domestic responsibilities in the community are executed by women. Some of the day-to-day housekeeping activities of young and older women include preparing food and feeding the family, taking care of children, taking cattle to farm/rearing, fetching and transporting water, grain grinding, collecting firewood, taking children to health facilities when they are sick, washing clothes, house making, and building houses, among others. The house building activity is very tiresome for women and also it is a continuous duty because of the mobile lifestyle of the community. Men’s primary occupation involved herding of goats and camels for most of the rural households in the area being studied, and women were never supported in housebuilding. 

Similarly, young women spend their time engaged in various activities including duties beyond their capacity. As a result, they lack time to even eat food and gain sufficient rest. Many girls in Afar do not believe young men could do their chores. On the contrary, young men spend their time socializing and sitting in the shade. A key informant from Awash Fentale Woreda said, “*being male is enough to live in this area without doing anything.*” Unlike young people in urban areas, adolescent women living in rural villages have more responsibilities compared to adolescent men. For young women, unlike young men, the burden of work increases after marriage (during marriage life). The responsibilities of younger and older men are limited to shepherding camels. According to a key informant interview from Amibara Woreda, the workload of women and men is incomparable. The most time-consuming work of women is collecting water, which is mainly attributed to the distance of the water source (rivers or springs) [15].

*“Our daily life revolves around cattle, goats, and herding. We do not have the time to properly eat. We eat our dinner in hurry and go to bed. The next day, after we pray (salawat), life continues-herding/rearing, searching for water, searching for pasture, and returning home in the afternoon. We do not have extra time to go to health facilities. We were born in the middle of the cattle, we live with them, and we die here.”*

—Woman, aged 30, Amibara District

Moreover, focus group discussion participants from Duelcha Woreda witnessed that compared to young men, young women take the responsibility of the house next to their mothers. Daily chores are as much a responsibility for younger women as they are for older women.

### 3.4. Access and Control over Resources or Assets

***Access to education****:* In Afar, most young women are encouraged to participate in domestic work or income-generating activities. The community does not send their daughters to school, as they want them to shepherd cattle, marry and bear children, and handle household work. Alternatively, their sons’ education is encouraged and accessible. Parents choose not to send their daughters to school unless they are forced or fined by the local administrative officials (most recent inconsistent practice of officials). If a mother sends her daughter to school, the daughter’s “Absuma” [cousin–prospective husband] can stop the daughter from continuing her schooling. Evidence suggests that the most common factors affecting young women’s education are busy domestic duties, the effects of early marriage by Absuma, and low household income and mobility due to the nature of activities in which they are engaged [20]. The Absuma tradition is arranged for daughters at birth to their eldest male cousin. Throughout the daughter’s childhood, her Absuma has the decision-making power about the fate of her education and sexual and reproductive health rights. 

***Access to and control over land, cattle, goats or camels****:* Younger and older women in Afar are a disadvantaged segment of the population in relation to property and asset ownership. Study participants stated that young women were not welcomed by the community to work and have their own wealth. In some cases, the only source of inherited wealth are gifts such as goats or cattle that they receive from their parents, as it is believed that young women do not need property prior to their marriage.

For young men, however, goats, camels and cattle are designated property for them from the time of birth. They use this inherited wealth to financially support their marriage. However, in some cases, both young men and women do not own any property. Parents own, sell cattle (resources) and cover education-related expenses for their children and are controlled by men. This is mainly rooted in the practice of dowry giving at the time of marriage. In Afar, customs affiliated with marriage are integrated with social and cultural norms. As a result, the community assumes that the man has power over resources and is considered the head of the household.

*“The community believes that a girl should not have to earn wealth because after she got married, she will go to her husband’s family or his clan. If she builds her own wealth, during marriage it is assumed that she may take her family wealth to another family or clan. That is why a female is not expected to earn wealth unlike boys.”*

—focus group discussion participant from Dulecha Woreda.

In Afar, women are unable to participate in business (sales or purchasing). Women own little to no assets and are discouraged from engaging in non-domestic activities. When marriage is proposed, the daughter’s parents count the number of cattle and camels owned by her “Absuma.” In most cases, men (husbands) own and control the most important assets of the households including land and livestock.

*“The number of camels, cattle, goats and sheep the household owns measure wealth in this area. …. Although some women may own these resources, men take higher proportion. [If divorce happens], a woman is expected to leave the house for the man. Similarly, a father gives high value to males than females in the household. Hence, he inherits his asset to his son than to his daughter.”*

—A key informant from Awash Fentale Woreda.

He added that today it is more common for adolescent men to earn their own income. They are working with various enterprises in areas of sugarcane development, fruit and vegetable agriculture, palm development and carpet works. However, access to income-generating activities and wealth is still very limited for women and youth girls in Afar. 

***Value given to younger and older women by the community****:* Societal values are held higher for younger and older men than they are for younger and older women and indeed, for non-married older women who are unable to reproduce. This is demonstrated by family responsibilities. Since resources are in the control of men, men are required to provide total support to their family if parents pass away. For example, if the parents of a given household pass away, male members of the household are responsible for the children or elders of the household, even if they are married and have children. Women with children are held in higher regard for societal values and respect in Afari communities due to the importance of marriage and child bearing in Afar.

*“If a woman is not able to give birth, no one wants to marry her and in fact her husband may divorce her. Since the culture encourages polygamy for males, even though he has multiple wives and if one of them is not able to give birth, he can divorce her and marry another woman. Here, marriage is established for to have a greater number of children not for love or life sharing.”*

—A focus group discussant from Dulecha Woreda.

Moreover, in Ethiopia, in particular in the Afar region, widows and divorcee women are considered bad luck and they are usually avoided. They are unwelcome at many social events, ceremonies and rituals and are stigmatized in many situations. The economic and social support that a widow receives from her late husband is typically extremely limited. A variety of customs and beliefs will prevail including privacy and confinement from family and community, a permanent change of diet and dress and discouragement of remarriage. As a result, women in this region avoid divorce and usually experience gender-based violence and inequality in their lives [4,5].

### 3.5. Decision-Making Autonomy and Women Empowerment

***Decision on marriage:*** One of the challenges for women in Afar is their low decision-making power both at home and within the community. Open discussion within the household and the extended family are much more restricted. The rigid culture and tradition of marriage makes it difficult for a young woman to marry a man of her choice. This is because nearly all marriages in the study area follow the “Absuma” procedure, i.e., daughters are only allowed to marry their eldest cousin. The Absuma makes marriage a ritual and prevents women from choosing their spouse. This lack of decision-making power prior to wedlock continues during the marriage life. Women are unable to freely exercise their sexual and reproductive health rights due to the traditional requirement of the man making the decisions. Alternatively, a young woman can marry another person if and only if her eldest cousin does not want to marry her [21,22]

*“When a girl is born in the household, the name of her Absuma (future prospect husband) will be written and kept in the household. It is immediately after birth that the family arranges the husband for the new baby girl. The girl will definitely marry the man arranged for her [when she is ready].”*

—focus group discussion participant, a girl from Amibara Woreda.

Fathers and mothers have limited say on the marriage prospect and process of their daughters in the Absuma tradition. The decision to have a sexual relationship before marriage or to marry a man of her choice is left to the cousin and the daughter’s uncle or cousin. It is considered taboo for a young woman to propose marriage to another man. However, if another man wants to marry a young woman, he must initially gain permission or approval from her uncle or cousin (Absuma). Residents must abide by “Omani” which is the custom of paying fines for marrying a man other than her cousin. Even if a young woman refuses to marry her cousin, she may be forced by the community, her uncle, or the clan leader to marry him. This situation results in many women fleeing their village to avoid offending the community and allay the criticism and stigma directed towards her parents. These young women migrate to faraway places to avoid undesirable marriages and to attend school or work.

*“If a girl wants to get married with whom she loves and not with her ‘Absuma,’ both she and her boyfriend will move to another area, most of the time to Djibouti, and they live there together.”*

—A key informant from the Awash Fentale Woreda Social Affairs Office.

If a mother does not want her daughter to get married against her will, the mother will be penalized by her tribe. She can be forced to bring her daughter and give her away to the man who proposed. If she fails to do so, she will be isolated from the community. The “Absuma” decides whom she will marry. On the other hand, if a girl becomes pregnant without a legal marriage, the culture encourages her to marry the man who impregnated her. The community will conduct a “Nikah” and force them to marry. If she undergoes a termination of pregnancy, which is not accepted in Afar, the boy will be punished and will be forced to marry her or distance himself from her. The girl may be stigmatized and isolated, but her parents are typically blamed by the community for not raising her “properly.” 

***Decision-making on Health services and FGM practices****:* In Afar, parents generally have the decision-making power about whether their daughter undergoes FGM; particularly the father and male relatives. It is practised at an early age before celebrating the first anniversary of the child. A woman cannot use any contraception without the knowledge and agreement of her husband. If her husband finds she is using contraceptives without his knowledge, he may quarrel with or beat her and the health professional who provided the service. It is also considered taboo for a man to use a condom and may result in him being isolated by the community. When a woman or her children get sick, she may not be able to see a doctor in a timely manner unless her husband decides. She also may not receive adequate delivery of quality services and medication. She cannot access facilities unless her husband agrees.

*“Still the most important decision on SRH including giving birth at health center, planning when to have the next child, etc., depends on the man [husband]. If the husband does not give permission, the midwife does not attend/assist during delivery. The same is true in the case of family planning. Since most husbands do not allow the use of contraceptives, interested women use in secrete without the knowledge of their husbands.”*

—Key informant interview from Awash Fentale Health Center

### 3.6. Younger and Older Women’s Sense of Self-Efficacy and Capability to Make Life Choices

We examined the level of awareness, attitude and practices of women, adolescents and youth about gender-related roles and responsibilities, their sexuality and level of participation in decision-making processes in relation to sexual and reproductive health rights, and their life choices. According to the findings of this study, there are low levels of awareness on reproductive health issues, especially among women. Many women accept their roles and responsibilities embedded in their culture and tradition and do not believe that men should share women’s duties.

Sexual identities and relationships are not openly discussed among communities in Afar, even amongst couples in most areas in Ethiopia. Sexual intercourse before marriage or extra-marital sexual relationships are considered taboo for women. Parents feel ashamed and consider these acts disrespectful if daughters engage in these activities. Sexual relationships are typically decided by the men in the community. The “Absuma” tradition provides more freedom to young men rather than young women and prevents them from initiating sexual relationships.

*“Men can marry other than their Absumas but girls are expected to marry their Absuma. Even if her Absuma marries another girl, another Absuma will be arranged for her. If she tries to marry a person other than her Absuma, the non-Absuma husband is expected to pay huge amount of compensation, which most do not do. Overall, it is not good for her.”*

—Schoolboy focus group discussion participant from Awash Fentale Woreda.

It is not socially acceptable for younger and older women to freely engage in sexual relationships in comparison to males. Sexual activity is traditionally viewed as a male-initiated act and an act only desired by men. A key informant from Awash Fentale shares this idea as follows:

*“Traditionally, couples do not even sleep together/share a bed. The man sleeps alone while the woman sleeps with her children. If the man has a sexual craving, he touches his wife with stick and she will join him. After intercourse, she gets back to her children and sleeps there….a woman [in Afar] engages in sexual intercourse only to have a child but not to satisfy her sexual desire.”*

### 3.7. The Effects of Gender Inequality in Women’s Sexual and Reproductive Health

According to Ethiopian Demographic and Health Survey (EDHS) reports, teenage pregnancy or childbearing is defined as women aged 15–19 years of age who have birthed or are pregnant with their first child, and this poses a critical health problem. It is associated with higher morbidity and mortality for both the mother and the child. The region has the highest teenage childbearing rates in Ethiopia (23%). Moreover, teenage pregnancy rates have not improved since 2016 (23%) compared to the 2000 EDHS rate (21%) [4]. Additionally, early childbearing has impacted on the social status of adolescents and youth mainly related to educational attainment due to school attrition rates [4]. In this study, the major problems mentioned by both the focus group discussion participants and key informants were related to early marriage, FGM, family planning, skilled birth attendants and termination of pregnancy, amongst others. 

The nature of marriage in the Afari community is more than just arranged marriages. Matrimony is arranged or known even before birth, which serves as a bond within but not between families due to the Absuma tradition. According to the Afari custom, daughters marry their maternal cousins [21]. Mostly, Absuma is arranged through the daughter’s mother’s line. Hence, if the mother identifies as a non-Afari ethnic group, the daughter may not be subject to Absuma. A focus group discussion participant from Amibara stated, “*I don’t have Absuma, my mother is Oromo.*” 

According to the 2016 EDHS report, in Afar, for the majority of the time (82%) parents or the community make the decisions about a woman’s first marriage and that is automatically tied to the Absuma norm. Although early marriage is strictly forbidden by law, it is still one of the problems especially in the rural parts of Afar and one of the causes of interruption for young women’s schooling. Moreover, women frequently experienced gender-based violence, and acceptance of wife beating by women in the region is as high as 69% [23,24]. FGM/C remains widespread across the region with the majority (91%) of younger and older women aged 15–49 years cut before the age of one year in more than half (52.5%) of females [4]. The misconception among men is an existing challenge to change people’s attitude towards FGM practices in the region. Men are pro-FGM at least to a certain extent. A male focus group discussion participant from Awash Fentale Woreda expressed his views as follows:

*“Yes, we [men] do not want FGM practice to be totally avoided or stopped. There is a type of FGM that we want to be practiced. We don’t want the previous type. The previous one involves complete removal of the flesh [of the genitalia]. What we want is this [he showed his thumb up] should be cut like that of the boys. Cutting that part is a norm. We want that to be cut. Can both the man and the woman have penis? If not circumcised, that [the clitoris] of females is also like a penis. So, we want it cut. Previously, they remove the whole flesh and then they tie their legs. The girl has to sleep for a week. She cannot take bath so it can stick together [get stitched]. We don’t want that, no more. We don’t want that to be totally practiced. But we want it to be cut in small portion.”*

In terms of health service utilization, Afar is one of the regions that has the lowest maternal health service utilization in the country. For instance, the skilled birth attendant rate is 16%, which implies that 84% of home births are without an accredited health care provider. Our qualitative study shows that one of the major challenges towards improving service utilization is the critical value given to the cultures and norms. One of the main reasons why many women do not attend an institution to birth is that women do not want to be touched by male service providers, which is attributed to religious beliefs or the opposition from their husbands. In Afar, many men and women have negative attitudes towards family planning practices and men never use condoms. The community’s explanation for not allowing use of contraception is that they want to increase the population size of their tribe because they believe that they are surrounded by enemies from different tribes outside their region. Hence, children are important assets in the Afar community and the members of the community in general want to have as many children as possible. Religion is also another reason for not using contraceptives.

*“Still the most important decision on SRH including giving birth at health center, planning when to have the next child, etc., depends on the man. If the husband does not give permission, the midwife does not attend/assist during delivery. The same is true in the case of family planning. Since most husbands do not allow the use of contraceptives, interested women use in secrete without the knowledge of their husbands.”*

—key informant interview from Awash Fentale Health Center.

*“The burden of activities on woman has an influence on reproductive maternal and child health service utilization. There are women who come to the health center after missing their scheduled appointment due to their over burden with activities.”*

—key informant interview, Amibara Woreda.

The misconceptions around long-term use of contraceptives is another reason for the limited use of contraceptives. According to a key informant from Amibara health center, many women commonly think that long-term contraceptives lead to excessive bleeding during pregnancy. There is no open discussion about reproductive health and sexuality between couples. Women are reticent to share their health problems with their husbands. Overall, contraceptive use is considered taboo in the community.

*“Family planning users become weak and unable to withstand our climate [hot weather] here. They easily get tired. The family planning requires taking good food like eating meat and drinking milk every day that we cannot afford now days.”*

—Male focus group discussion participant from Awash Fentale Woreda

Women’s reproductive issues place a burden on the domestic house duties. The extra time taken to attend scheduled visits at health centres is challenging with their home duty commitments.

*“We do not have any spare time, and we are more burdened with our household activities than men. There is a challenge to go to the Health Centre anytime we want due to the burden we have with our household chores, it is difficult to use the services as needed. There are women in our community who go to the Health Centre anytime they want from the support they get from their children. They are very advantageous, they have given birth early and now they are getting this support.”*

—Focus group discussion participant, female youth.

### 3.8. Policy Implication

Findings reported in this study provide vital evidence to inform policy and decision makers to respond and prevent violence in alignment with the SDG’s target by 2030. The silence over the effects of gender inequality in the Afar region and in other developing regions of the country should be addressed by government and community advocacy campaigns. Many community members still do not have a deep understanding of the benefits of eradicating GBV and the occurrence of many practices and gender inequality such as FGM, widows’ poor financial situations and other harmful practices for younger and older women.

The government in this region needs to work in a unified way to target legal practices and policies in order to eradicate women’s poor decision-making capacity and reduced empowerment. Wealth distribution laws should be the first priority so that communities are not disadvantaged in their capacity to earn good incomes. Cultural ideas, norms, and customs are deeply embedded in the societal structure and men and religious leaders should be educated to ensure that individuals continue to live their lives to ensure economic and social rights are upheld. The current Ethiopian Health extension programs may provide key education components so that each and every household will support women in the community. These strategies should be supported by a legal framework to accommodate social support that includes educational and economic growth and provision of health information and services for women and communities. 

## 4. Conclusions

In Afar, gendered division of labour within households and communities is shaped by the economic and social structures that have been deeply rooted over generations. Younger and older women in Afar participate in mostly unpaid, domestic and external responsibilities in comparison to younger and older men. Gendered formal and informal tasks of young women are more or less equivalent to that of older women in Afar in many ways. Women are responsible for a majority of the laborious activities in the community, and some of the day-to-day activities of young and older women exclude them from public or community-level engagement. Daughters spend most of their time engaged in different activities including duties beyond their capacity. They seldom take breaks to rest, even during pregnancy, working up to the date of their birth. Alternatively, work expectations are lower for younger and older men as their primary role is shepherding camels.

According to the findings of this gender analysis, younger and older women have low control over resources at the household and community levels. Schooling is a priority for sons while daughters are encouraged to marry early in accordance with the cultural process of Absuma. This limits young women’s access to education and this extends to access to property and asset ownership. Women are unable to acquire their own capital and most family assets are under the control of men, and in many cases, women are not permitted to make decisions to sell or buy on behalf of the family.

Existing social and cultural norms, including Absuma, prohibit women and young people from having decision-making power. Instead, men, parents, and other elders possess total decision-making power. This prohibits women and young people’s access to education and financial and material resources. It also places direct control of capital in the possession of men. The study concludes that adolescents and youth have limited participation in decision-making processes at household and community levels. 

Sexual reproductive health service utilization for women in Afar is poor for different reasons including high workload, poor awareness, low accessibility to specialized services (hospitals), lifestyle (mobility), and cultural/religious factors, and this continues to contribute to the dominance of social norms, namely the patriarchy over sexual and reproductive health issues faced by this population. For those women who were aware of sexual reproductive health services, they maintained the status quo due to other commitments. Genital cutting practices, child marriage and gender-based violence are the most prevalent issues in the region. Harmful traditional practices in Afar can be punishable by law and strengthening the programmatic efforts of different sectors will help to reduce the problem. Enforcement bodies subsequently respond appropriately to protect the rights of women; however, this enforcement is not practiced consistently and widely.

Generally, the gender imbalance has a major impact on the health of younger and older women, and national laws related to their reproductive health rights are not respected or implemented as expected. Designing a coordinated intervention is recommended as a priority for governments and key stakeholders. This intervention could be targeted at priority areas including gender inequalities, improving young women’s education, family planning and reproductive choices, social and behavioral communication, and restricting early and harmful marriage practices.

## Figures and Tables

**Table 1:** Demographic background of focus group discussion participants by Woreda, December 2019.

Name of Woreda	No.	Group Type	Number of Focus Group Discussion Participants
Amibara	1	Married women (aged 20–29 years)	11
	2	Never married (10–14) in-school rural girls	6
Argoba	3	Married adult women (aged 30–49 years)	12
Awash Fentale	4	Adult men (community members) aged 30–54 years	13
	5	Adult women (community members) aged 20–29 years	10
Awash town	6	Never married in-school boys aged 15–19 years	8
Duelcha	7	Out-of-school girls aged 20–29 years (married only groups)	9
	8	10–14 years out-of-school boys	12
